# Meta-Analysis on the Relationship between HLA-DRBl Gene Polymorphism and Cervical Cancer in Chinese Population

**DOI:** 10.1371/journal.pone.0088439

**Published:** 2014-02-14

**Authors:** Lin-zhen Wei, Hai-lin Wang, Xin Liu, Ya-peng Lu, Fei Xu, Jin-qiu Yuan, Ya-qin Ling

**Affiliations:** 1 Department of Obstetrics and Gynecology, Gansu Provincial Hospital, Lanzhou, Gansu, China; 2 The First Clinical Medicine College of Lanzhou University, Lanzhou, Gansu, China; 3 Institute of Pathogenic Biology, School of Basic Medical Sciences, Lanzhou University, Lanzhou, China; 4 Division of Epidemiology, School of Public Health and Primary Care, The Chinese University of Hong Kong, Hong Kong; University of Cape Town, South Africa

## Abstract

**Aim:**

To determine the association between HLA-DRB1 haplotypes and risk of cervical cancer in unselected and samples from Chinese ethnicities.

**Methods:**

A comprehensive search for articles from their inception to April 1st, 2013 was conducted from PubMed, Medline, Elsevier Science, Springer Link, Cochrane Library database, China biology medical literature database (CBM),China National Knowledge Infrastructure (CNKI),VIP,and Chinese literature database(Wang fang). A total of 1596 patients with cervical cancer and 2048 controls from the 12 studies on the relationship between gene polymorphism of HLA-DRB l and cervical cancer were performed and data were analyzed and processed using Review Manager 5.0 and Stata 11.0.

**Results:**

Among the 13 family alleles, two (DRB1*03 and DRB1*08) were found to be negatively associated with cervical cancer in all studies or in Uighur subgroups, and two (DRB1*10 and DRB1*15) were positively associated with in all studies or in Uighur subgroups. Among the 25 specific alleles, six (DRB1*0301, *0403,*0404, *0803, *1312 and *1502) were associated with an increased risk cervical cancer in all studies. No significant association was established for other HLA-DRB1 family alleles and specific alleles. Ethnicity partially explained the race influence of DRB1*12, DRB1*14, DRB1*0301, DRB1*0403, DRB1*0404, DRB1*0803, DRB1*1312 and DRB1*1502 phenotypes.

**Conclusion:**

Our results support the hypothesis that the HLA-DRB1 family alleles and specific alleles might influence the susceptibility or resistance to cervical cancer, suggesting that immune regulation may play a key role in this disease, although further investigations are still needed.

## Introduction

Cervical cancer is one of the leading causes of cancer-related death among women worldwide, with 88% of cases occurring in less developed countries [1]. It is a grave health problem in China, with 132,300 women developing the disease each year, and is the second cancer in Chinese women [2]. Whereas a number of factors have been implicated in the etiology of cervical cancer, there is ample epidemiological and clinical evidence supporting that persistent infection with oncogenic types of human papillomavirus (HPV) predisposes to the disease, with the contribution of additional co-factors such as smoking and oral contraceptive use. A strong association exists between persistent HPV infections and risk of cervical lesions, especially for HPV types 16 and 18 [3]. Intriguingly, HPV infection is necessary but not sufficient to induce cellular abnormalities and the development of invasive cancer, since prospective studies consistently show that only a small fraction of infected women do eventually develop the disease [4]. A comprehensive study indicated that HPV interacts with other cofactors, including HLA class II alleles [5], that influence the risk of HPV persistence and progression to cervical cancer.

Human leukocyte antigens (HLA) comprises a family of Class I and Class II genes within the major histocompatibility complex, which is located on the short arm of chromosome 6 (6p) in humans [6]. HLA Class II genes encoded by DR, DQ, and DP genes are expressed in immune cells and are of importance in the regulation of the immune response to foreign antigens and discrimination of self from non-self antigens [7], [8]. They present antigenic peptides to specific T-cells to initiate a cell-mediated immune response to HPV infection.

The etiology of cervical cancers might be related to risk factors, and HLA-DRB1 gene polymorphism was initially proposed in the late 1990s and has created considerable interest [9]. Findings from published studies that have examined the association HLA-DRB1 alleles and the risk of cervical cancer have been inconsistent. Several studies have reported a positive relation between HLA-DRB1 alleles and cervical cancer, but findings in different ethnic population have been controversia [10]–[12]. As many conflicting reports have been relatively small in sample size, we performed a meta-analysis that examined the association between HLA-DRB1 allele families and alleles and cervical cancer. Our purpose was to evaluate the evidence from studies on genetic basis and the risk of cervical cancer by summarizing it quantitatively with a meta-analytic approach, and to find evidence for the prevention and intervention of cervical cancer.

## Methods

### 1. Literature and Research Strategy

Studies published in English and Chinese were considered in this study. Studies in English were identified through PubMed, Medline, Elsevier Science, Springer Link and Cochrane Library database from their earliest available date to April 1st, 2013. Reports in Chinese were found through China National Knowledge Infrastructure (CNKI) (1979–April 1st, 2013), Database of Chinese Scientific and Technical Periodicals (VIP) (1989–April 1st, 2013), Chinese literature database(Wan fang) (1986–April 1st, 2013) and China biology medical literature database (CBM) (1970–April 1st, 2013). Key words (“cervical cancer” OR “cervical carcinoma” OR “uterine cervical carcinoma”) and (“HLA-DRB1” or “human leukocyte antigen” or “HLA antigen”) were used in combination to retrieve the relevant literatures in all these databases. Moreover, we reviewed the reference lists from retrieved articles to search for further relevant studies. This Meta-analysis was planned, conducted, and reported in accord with standards of quality for reporting meta-analyses [13].

### 2. Inclusion and Exclusion Criteria

The inclusion criteria were: (1) studies presented original data and the number of genotype of HLA-DRB1 in cases and controls; (2) the articles provided raw data including odds ratio (OR) with 95% confidence interval (CI) and respective variance, or the relevant information could be calculated; (3) analytical study (case -control study or cohort study) or experimental study; (4) the diagnosis of CC was based on at least one of the following criteria: typical histological characteristics or colposcopy biopsies.

The exclusion criteria were: (1) raw data not available for retrieval; (2) repetitive reports (If data were duplicated in more than 1 study, we included the study with the largest number of cases.); (3) the study did not fit the diagnosis criteria.

The frequency of HLA-DRB1 alleles varies according to ethnic and racial background, with some alleles being extremely rare. Therefore, articles were not required to identify all alleles for inclusion.

### 3. Data Extraction

To decide inclusively or exclusively, articles were identified by two independent reviewers using a standardized data extraction form designed by our group. Data with discrepancies in identification were discussed. If consensus was not achieved, the decision was made by a third reviewer. The following data were extracted from each study: the first author’s name, publication year, area where the study was performed, study period, range of age, number of cases and controls, HLA-DRB1 type alleles, diagnostic method, control sample description (if there was more than one control group, we choose the healthy group as the control group in order to minimize the confounder). The main features of the trials included in the meta-analysis are shown in Table 1.

**Table 1:** Characteristics of studies included in the meta-analysis.

Authors and year of publication	Area and Nation	Number of cases			Number of controls			Number of DRB1 alleles studied	Diagnostic methodof CC	Detection Methods	Control Type
		NO(%)	Mean age±SDor (range)		NO(%)	Mean age±SD or (range)					
Yuh-Cheng Yang et. al [17],2006	Taiwan ND	126 50.1±12.5		(25.2–89.6)	289 40.1±4.8		(31.7–46.5)	32	Pathology	PCR-SBT	Healthy
Du Yang et. al [18],2004	Liaoning ND	43		47 (25–76)	58		44 (22–62)	13	Pathology	PCR-SSP	Healthy
Guzalnur.Abliz et,al [19],2008	Xinjiang Uighur	200		45.5(23–76)	200		44.3(21–66)	13	Pathology	PCR-SSO	Benign lesions
Paul K.S. Chan et,al [20],2005	Hong Kong	173		45.6(20–82)	323		36.8(25–56 )	13	Colposcopy biopsies	PCR-SSO	Blood donors
Yangguifang et,al [21],2009	Tianjin ND	30		37(ND)	66		ND(45–60)	13	Pathology	PCR-SBT	Uterine fibroids
Suqi et al [22],2009	Xinjiang Uighur	192		ND	203		ND	1	Pathology TBS	PCR-SSP	NILM
	Xinjiang Han	95		ND	94		ND	1	Pathology	PCR-SSP	Benign lesions
LuLing et al [23], 2008	Xinjiang Uighur	300		45.4(23–76)	300		43.8(21–66)	13	Pathology	PCR-SSO	NILM benign lesions
LiHua et al [24], 2010	Xinjiang Uighur	90		45.9(21–76)	90		43.2(21–66)	13	Pathology	PCR-SSO	NILM benign lesions
Mirijili Jilili et al [25], 2009	Xinjiang Uighur	92		44.5(23–72)	92		42.8(21–66)	13	Pathology	PCR-SSO	NILM benign lesions
HuangJinshuang et al [26],2007	Liaoning ND	53		ND	34		ND	6	Pathology	PCR-SSP	Uterine fibroids
Yuping Wu et al [27], 2007	Han	133		46.7±11.4	98		40.5±8.5	17	Pathology	PCR-SBT	Healthy
M Zhao et al [28], 2012	Mid-wesrern China ND	69		ND	201		ND	17	Pathology	PCR-SBT	Healthy

ND, not described.; PCR-SSO, PCR-sequence-specific oligonucleotides. PCR-SBT, PCR- sequence-based typing; PCR-SSP, PCR- sequence-specific primer; NILM-Negative for intraepithelial lesion or malignancy.

### 4. Statistical Analysis

(1) The pooled OR and 95% CI were determined by Z test with P<0.05 considered statistically significant; (2) Heterogeneity across studies was estimated using the Cochran’s Q statistic and I^2^ test. Meta analysis was carried out by using random-effects or fixed effects model based on the pooled effect estimates in the presence (p≤0.1 or I^2^≥50%) or absence (p>0.1 or I^2^<50%) of heterogeneity [14]. (3) To assess the presence of publication bias statistically, Begg’s test and Egger’s regression test were preformed where there were three or more studies [15]–[16]. p<0.05 was considered representative of statistically significant publication bias. (4) In this meta-analysis, in order to better investigate possible reasons of between-study heterogeneity, Studies were categorized into subgroups based on ethnicity status.(5)All analyses were performed using Review Manager 5.0 and Stata 11.0. All the P values were two sided.

## Results

### 1. Study Selection and Study Characteristics

**Figure 1:**
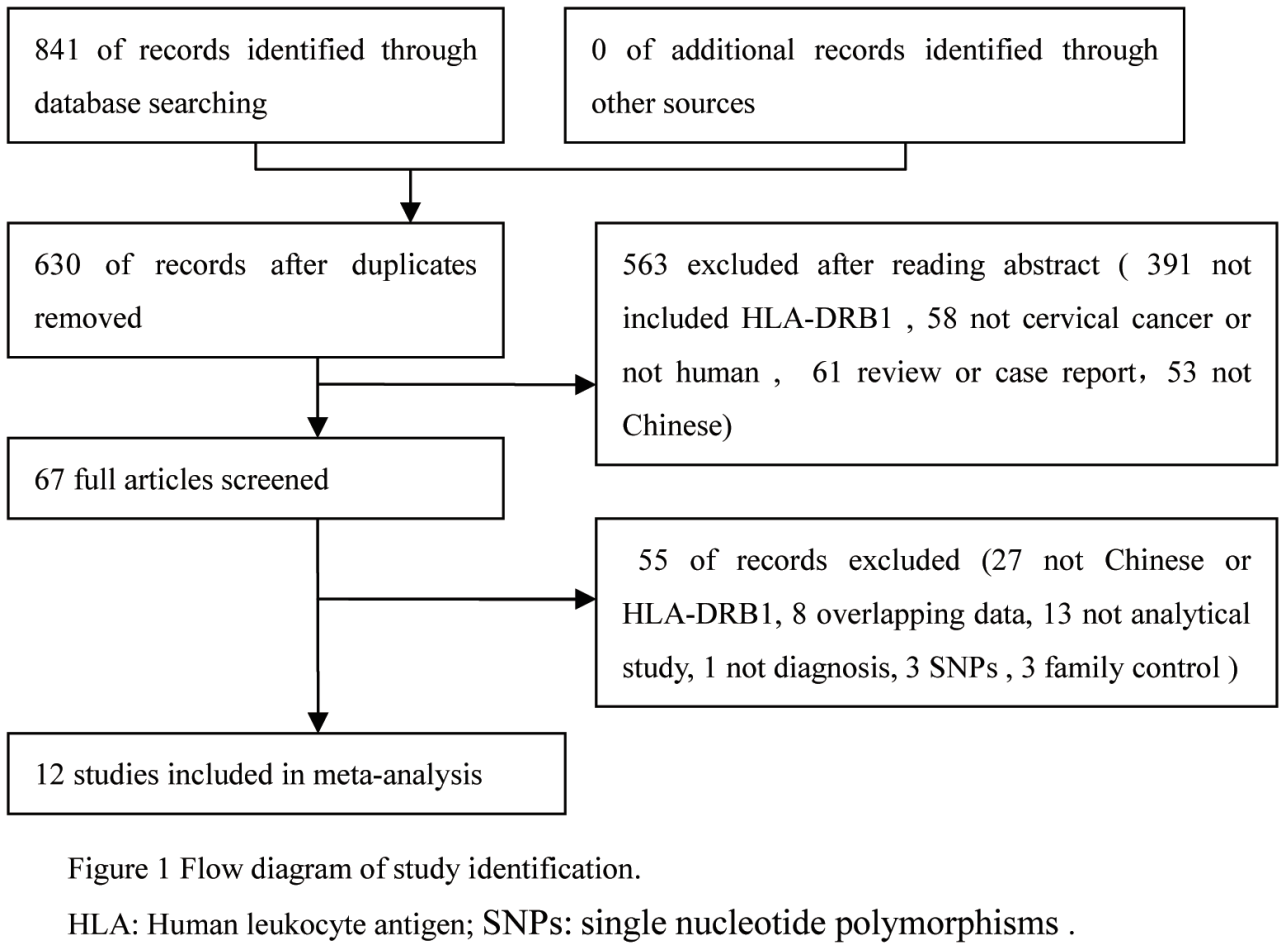
Flow diagram of study identification.

After carefully reading each article, 12 studies were eligible for the meta-analysis (including 5 in English and 7 in Chinese). A flow diagram of the study selection process is shown in Figure 1. We identified a total of 841 potentially relevant articles to our search criteria, of which twelve studies examining the association between HLA alleles and cervical cancer are presented in Table 1. All the selected studies presented original data on independent samples. A total of 1596 patients with cervical cancer and 2048 controls were included from 12 studies. Five studies used PCR-sequence specific primer for HLA, while others used PCR- sequence-based typing or PCR-sequence specific oligonucleotide for HLA.

### 2. Meta-analysis Results

A summary of the meta-analysis findings between HLA-DRB1 genetic polymorphisms and susceptibility to cervical cancer worldwide was provided in Table 2. In total, only thirty-eight HLA-DRB1 alleles from these studies were included in the meta-analysis, and 15 specific alleles were excluded because each was identified in only one study. A total of 13 DRB1 allele families and 25 specific alleles were extracted from the studies to investigate their association with cervical cancer.

**Table 2:** Meta-analysis of associations between HLA-DRB1 alleles and cervical cancer.

Alleles	No. of study	Case	Control	heterogeneity P value	I^2^ value (%) for heterogeneity test^+^	Model	OR(95%CI)	P value	Z	P value for Egger’s (Begg’s) bias test
01	8	84/1077	79/1389	0.16	34%	F	1.04(0.75∼1.43)	0.83	0.21	0.983(0.711)
03	7	172/1024	272/1352	0.27	21%	F	0.74(0.59∼0.91)	0.005	2.82	0.986(1.000)
04	7	215/967	304/1264	0.03	58%	R	0.94(0.67∼1.31)	0.72	0.36	0.582(0.548)
07	6	314/898	315/1063	0.48	0%	F	1.08(0.88∼1.32)	0.46	0.74	0.821(0.707)
08	7	87/967	183/1264	0.12	41%	F	0.68(0.52∼0.90)	0.006	2.73	0.655(1.000)
09	6	139/898	175/1063	0.73	0%	F	1.10(0.86∼1.42)	0.46	0.74	0.849(1.000)
10	6	43/898	22/1063	0.92	0%	F	2.30(1.37∼3.86)	0.002	3.14	0.278(0.707)
11	8	194/1020	268/1298	0.001	71%	R	0.89(0.58∼1.37)	0.60	0.52	0.136(0.266)
12	5	77/898	162/865	0.83	0%	F	0.63(0.46∼0.87)	0.005	2.78	0.484(0.707)
13	8	171/1020	207/1298	0.04	52%	R	0.88(0.70∼1.11)	0.28	1.09	0.490(0.386)
14	6	100/898	92/1063	0.53	0%	F	1.39(1.03∼1.88)	0.03	2.15	0.139(0.133)
15	10	362/1307	307/1595	0.0007	69%	R	1.62(1.36∼1.93)	<0.00001	5.33	0.582(0.474)
16	5	17/898	47/1063	0.95	0%	F	0.56(0.32∼1.00)	0.05	1.97	0.254(0.086)
0101	3	6/225	15/393	0.64	0%	F	1.03(0.40∼2.67)	0.94	0.07	0.316(0.296)
0301	2	4/156	23/355	0.07	69%	R	4.36(1.15∼16.48)	0.03	2.17	NA
0401	2	4/156	3/355	0.28	16%	F	3.05(0.67∼13.84)	0.15	1.45	NA
0403	2	18/156	15/355	0.37	0%	F	2.98(1.47∼6.04)	0.002	3.04	NA
0404	2	9/156	4/355	0.85	0%	F	5.06(1.62∼15.79)	0.005	2.79	NA
0406	2	14/156	8/355	0.02	82%	R	1.82(0.05∼72.72)	0.75	0.32	NA
0701	3	18/225	39/556	0.28	22%	F	1.14(0.63∼2.06)	0.67	0.43	0.469(0.296)
0802	2	4/156	4/355	0.07	70%	R	5.24(3.08∼8.91)	0.70	0.38	NA
0803	2	51/156	31/355	0.31	2%	F	5.27(3.19∼8.70)	<0.00001	6.48	NA
0901	4	121/278	151/590	<0.00001	97%	R	0.93(0.008∼10.48)	0.95	0.006	0.159(1.000)
1001	4	26/278	19/590	0.13	47%	F	1.83(0.93∼3.60)	0.08	1.75	0.471(0.734)
1101	2	55/156	31/355	0.01	83%	R	4.21(1.03∼17.25)	0.05	2.00	NA
1104	2	1/156	1/355	0.33	0%	F	2.23(0.31∼15.94)	0.42	0.80	NA
1201	3	33/225	55/556	<0.00001	93%	R	1.48(0.14∼15.63)	0.74	0.74	0.818(1.000)
1202	2	79/156	33/355	<0.00001	95%	R	2.11(0.02∼260.74)	0.76	0.30	NA
1301	3	2/225	6/556	0.38	0%	F	1.09(0.27∼4.32)	0.91	0.12	0.537(1.000)
1302	2	15/156	10/355	0.01	84%	R	1.50(0.03∼68.16)	0.83	0.21	NA
1312	3	6/250	1/453	0.99	0%	F	6.38(1.28∼31.69)	0.02	2.26	0.503(1.000)
1401	2	22/156	7/355	0.04	75%	R	5.11(0.60∼43.31)	0.13	1.50	NA
1404	2	1/156	2/355	0.22	35%	F	1.64(0.11∼25.51)	0.72	0.35	NA
1405	4	16/319	20/654	0.007	75%	R	1.15(0.16∼8.28)	0.89	0.14	0.330(1.000)
1407	2	1/156	2/355	0.23	30%	F	1.50(0.25∼9.10)	0.66	0.44	NA
1501	2	53/156	37/355	0.003	89%	R	2.90(0.51∼16.43)	0.23	1.20	NA
1502	2	20/156	22/355	0.35	0%	F	2.26(1.18∼4.33)	0.01	2.45	NA
1602	2	26/156	11/355	0.14	55%	R	4.12(0.71∼24.49)	0.11	1.58	NA

Heterogeneity is present when p values less than 0.1 or I^2^values equal or more than 50%.

Three allele families (DRB1*10, *14, and *15) conferred a significantly increased risk and four allele family (DRB1*03, *08, and *12) conferred a significant protective effect for cervical cancer. Their combined OR value, 95% CI and Begg’s and Egger’s tests are listed in Table 2. These results suggest that patients with DRB1*10, DRB1*14 and DRB1*15 alleles were at a higher risk of developing cervical cancer than those with DRB1*03, *08, and *12 alleles. Through comparison of the HLA-DRB1 alleles frequency in control groups between different studies in the genotyping level, we found that there was no significant difference between control groups in different studies in the following alleles: DRB1*01, DRB1*03, DRB1*07, DRB1*08, DRB1*09, DRB1*10, DRB1*12, DRB1*14, DRB1*16 (P>0.1 and I^2^<50%). On the other hand, there was a significant difference between different studies in control groups in the following alleles: DRB1*04, DRB1*11, DRB1*13, DRB1*15 (P≤0.1 and I^2^>50%). These analyses were based on the data from 12 studies irrespective of the ethnicity of the study populations. Publication bias in the studies was assessed by Begg’s and Egger’s tests, results showed that there were no evidence of publication bias (Table 3 ).

**Table 3:** Meta-analysis of relationship between HLA-DRB1 allele polymorphism and cervical cancer in the Chinese Uighur population.

Alleles	No. of study	Case	Control	heterogeneity P value	I^2^ value (%) For heterogeneity test^+^	Model	OR(95%CI)	P value	Z	P value for Egger’s (Begg’s) bias test
01	4	69/682	55/682	0.84	0%	F	1.28(0.89∼1.86)	0.19	1.32	0.590(0.734)
03	4	123/682	42/682	0.66	0%	F	0.62(0.48∼0.81)	0.0004	3.57	0.970(1.000)
04	4	142/682	170/682	0.04	0%	R	0.79(0.50∼1.24)	0.30	1.04	0.744(1.000)
07	4	294/682	273/682	0.70	0%	F	1.14(0.92∼1.41)	0.25	1.15	0.297(0.089)
08	4	42/682	76/682	0.75	0%	F	0.52(0.35∼0.77)	0.001	3.24	0.696(1.000)
09	4	73/682	71/682	0.58	0%	F	1.03(0.73∼1.46)	0.86	0.18	0.621(0.737)
10	4	33/682	15/682	0.83	0%	F	2.22(1.21∼4.10)	0.01	2.56	0.159(0.308)
11	4	139/682	199/682	0.01	74%	R	0.63(0.37∼1.06)	0.08	0.75	0.993(0.734)
12	4	32/682	43/484	1.00	0%	F	0.73(0.42∼1.27)	0.26	1.12	0.662(0.734)
13	4	112/682	126/682	1.00	0%	F	0.87(0.66∼1.15)	0.32	1.00	0.063(0.089)
14	4	71/682	50/682	0.93	0%	F	1.47(1.01∼2.15)	0.05	1.99	0.895(0.734)
15	5	247/874	162/885	0.08	52%	R	1.77(1.26∼2.50)	0.001	3.29	0.800(0.806)
16	4	5/682	8/682	0.94	0%	F	0.62(0.20∼1.91)	0.41	0.83	0.001(0.296)

F: Fixed effect model (Peto Mantel-Haenszel); R: Random effect model (Dersimonian-Laird).

Among the specific alleles, 6 (DRB1*0301, *0403,*0404, *0803, *1312 and *1502) were significantly associated with an increased risk, However, there are no specific alleles with a decreased risk (Table 2). We found that there was no significant difference between the control groups of the various studies for following alleles: DRB1*0101, DRB1*0401, DRB1*0403, DRB1*0404, DRB1*0701, DRB1*0803, DRB1*1001, DRB1*1104, DRB1*1301, DRB1*1312, DRB1*1404, DRB1*1407, DRB1*1502 (P>0.1 and I^2^<50%). However, there was a significant difference between different studies in control groups in the following alleles: DRB1*0301, DRB1*0406, DRB1*0802, DRB1*0901, DRB1*1101, DRB1*1201, DRB1*1202, DRB1*1302, DRB1*1401, DRB1*1405, DRB1*1501, DRB1*1602 (P≤0.1 and I^2^>50%). (Table 2). Begg’s and Egger’s tests showed that results no evidence of publication bias.

### 3. Subgroup Analysis

Since considerable diversity of ethnic groups existed among these studies, we performed further subgroup analysis based on ethnicity. Among studies in Uighur, two allele families (DRB1*04 and *08 ) were significantly associated with an decreased risk and two allele families (DRB1*10 and *15) were significantly associated with a increased risk for cervical cancer. Only DRB1*04 and DRB1*15 had heterogeneous (P≤0.1 and I^2^>50%), so a random effect was used. Begg’ s and Egger’s tests revealed no significant publication bias for any of the allele families (Table 3).

## Discussion

Genetic susceptibility to cervical cancer has been a research focus, and it has been discussed that the polymorphisms of a number immune response associated genes, including HLA-DR loci, affectes the susceptibility to and clearance of persistent HPV infection among different populations. HLA plays an essential role in the pathogenesis of HPV virus-associated cervical cancer. HLA-II genes are expressed as cell surface glycol-proteins that bind short peptide epitope to CD4+ T cells. HLA-DR, a subtype of HLA class II molecules, has a particular binding motif that dictates a specific range of peptides that can physically bind in a groove on the surface of the HLA molecule [29].

Cervical cancer is mostly a virus infected disease. However, the susceptibility of individuals with.persistent infection to develop invasive cervical cancer, even with the same HPV exposure, is varied. Host-factors, including gene polymorphisms, might be used to interpret these differences at least in part. [5]. Since many studies have revealed the relationship between HLA-DRB l gene polymorphism and cervical cancer in different populations, it is currently considered as a disease marker and contributes to the genetic risk.

However, recent studies on the association between HLA-DRB1 allele polymorphisms and cervical cancer have been inconclusive and controversial. Climent [12] reported that the DRB1*11 and DRB1*16 alleles might be risk factors for the occurrence of cervical cancer (OR = 2.89, OR = 1.74 respectively), DRB1*01, DRB1*04, DRB1*14, and DRB1*15 may be a protective allele (OR = 0.52, OR = 0.60, OR = 0.33 and 0.65, respectively). Yuh-Cheng Yang^17^DRB1*0701 and *1407 tended to confer a risk of CSCC (OR = 2.89 and 11.55, respectively). On the other hand, DRB1*1202 and *1401 played a major protective role in patients with cervical cancer (OR = 0.64 and 0.45, respectively). Furthermore, DRB1*15 was associated with susceptibility to cervical cancer in Swedish [11] and British [10] women, although several other studies, among American [30], French [31], and northwest English [32] women did not find an association between DRB1*1501 and cervical cancer.

Meta-analysis is a powerful method to increase the sample size from individual studies to enhance the statistical power of the analysis, which may reduce the probability that random error of producing false-positive or false-negative associations [33]. A total of 12 studies, including 3,410 cervical cancer cases and 1,735 healthy controls, were evaluated in the current meta-analysis, which addressed 38 HLA-DRB1 subtypes. The results showed that DRB1*03, *08,*11, and *16 were strongly related to cervical cancer as protective factors. Meanwhile, DRB1*10, *14, *15, *0301, *0403, *0404, *0803, *1312 and *1502 might be regarded as risk factors, but we could not gain significant results because *0301, *0403, *0404 and *0803 were only reported two papers. these results really require further studies. However, we could not find an association between special alleles with cervical cancer in Uighur population. Additionally, we found that DRB1*11, DRB1*0802 and DRB1*1405 in all groups and DRB1*11in subgroup have high heterogeneity but no evidence has shown the existence of publication bias.

A subgroup analysis was performed to evaluate the effects of ethnicity on the meta-analysis. When the six studies in Uighurs were analyzed separately (Table 3), 5 of 13 HLA-DRB1 allele families were found to be significantly associated with cervical cancer. Three allele families (DRB1*10, and *15) conferred susceptibility to cervical cancer and 2 allele families (DRB1*03 and *08) were protective. While such associations have previously been reported for DRB1*03, *08, *10 and *15, an association between HLA-DRB1*12,*14, and *16 alleles and cervical cancer in subgroup studies were inconsistent with previous one in all groups, which implies that some family alleles have a same effect between Uighur groups and other groups and some confer a genetic effect respective of ethnicity. One limitation for this meta-analysis is that not all alleles were reported in each Uighur study. Thus, one can not reach a conclusion that whether special alleles have a difference in the different groups through the meta-analysis.

Although the correlation of cervical cancer with HLA-DRB1 genes has been demonstrated by various studies, the mechanisms underlying the effect have yet to be elucidated. Human tumor cells express diverse types of antigens, depending on the etiology and pathogenesis of the disease [34]. Because tumor development is preceded by chronic inflammation, immune responses, whether towards the infectious agent itself or against tumor antigens, may be critical for development of tumor. HLA-DRB1 alleles may affect the way the human body involved in the immune system and in cell cycle [35]. Some alleles are considered protective while others increase the risk of developing a certain condition. Moreover, the same allele can be positively associated with a certain condition while it can be negatively associated with another.

This study also has its own limitations. First, since little literature including DRB1 genotype was available for inclusion in our meta-analysis, not all alleles were not reported in each Uighur study. This might can not reach a conclusion that whether special alleles have a difference in the different groups. Since only published studies written in English and Chinese were included in the meta-analysis, publication bias may occur. Secondly, although people in control groups were mainly healthy adults, there may be specific genetic effects among these controls and we could not entirely rule out the possibility of the incidence of cervical cancer in the future. It is necessary to use standardized unbiased methods on homogeneous cervical cancer patients and well matched controls. Third, our results were based on unadjusted estimates. A more precise analysis should be conducted with individual data, which would allow the adjustment by other co-varieties including age, ethnicity, family history, environmental factors and lifestyle. Finally, five studies used PCR-sequence specific primer for HLA, while others used PCR- sequence-based typing or PCR-sequence specific oligonucleotide for HLA at the time of the studies. The different typing methods were not identical between different laboratories, which might lead to a heterogeneity in current meta – analysis.

In summary, in the present study we performed a meta- analysis on the association of cervical cancer with the HLA-DRB1 alleles. Our results indicated the difference of HLA-DRB1 genetic susceptibility of cervical cancer in Chinese population. For the HLA-DRB1 family alleles polymorphism, a significantly association with cervical cancer was found in Chinese Uighur group, indicating that HLA-DRB1*03 and DRB1*08 alleles may be the protective factors for cervical cancer and HLA-DRB1*10 and DRB1*15 alleles may be the risk factors for cervical cancer, but not data found in other ethnic groups. Overall, ethnicity may play an key role in cervical cancer outcome. A major limitation of this pooled analysis of previously published data relies on the fact that several studies included limited sample size, so we can not get information of special alleles in Uighur group. More studies on individuals from various ethnic groups and large-scale and well designed case-control studies are needed to determine the role of HLA-DRB1 polymorphisms in the outcome of cervical cancer.

## Supporting Information

Checklist S1 **PRISMA checklist.**
(DOC)
